# CD4^+^ T cells with an activated and exhausted phenotype distinguish immunodeficiency during aviremic HIV-2 infection

**DOI:** 10.1097/QAD.0000000000001223

**Published:** 2016-09-28

**Authors:** Marcus Buggert, Juliet Frederiksen, Ole Lund, Michael R. Betts, Antonio Biague, Morten Nielsen, Johanna Tauriainen, Hans Norrgren, Patrik Medstrand, Annika C. Karlsson, Marianne Jansson, the SWEGUB CORE group

**Affiliations:** a Division of Clinical Microbiology, Department of Laboratory Medicine, Karolinska Institutet, Stockholm, Sweden; b Department of Microbiology, University of Pennsylvania, Philadelphia, PA, USA; c Center for Biological Sequence Analysis, Department of Systems Biology, Technical University of Denmark, Lyngby, Denmark; d National Public Health Laboratory, Bissau, Guinea-Bissau; e Instituto de Investigaciones Biotecnológicas, Universidad Nacional de San Martín, Argentina; f Department of Clinical Sciences Lund; g Department of Translational Medicine; h Department of Laboratory Medicine, Lund University, Lund; i Department of Microbiology, Tumor and Cell biology, Karolinska Institutet, Stockholm, Sweden.

**Keywords:** activation, CD4^+^ T cells, exhaustion, HIV-1, HIV-2, immunodeficiency, viremia

## Abstract

HIV type 2 (HIV-2) represents an attenuated form of HIV, in which many infected individuals remain ‘aviremic’ without antiretroviral therapy. However, aviremic HIV-2 disease progression exists, and in the current study, we therefore aimed to examine if specific pathological characteristics of CD4^+^ T cells are linked to such outcome.

**Design:**

HIV-seronegative (*n* = 25), HIV type 1 (HIV-1) (*n* = 33), HIV-2 (*n* = 39, of whom 26 were aviremic), and HIV-1/2 dually (HIV-D) (*n* = 13)-infected study participants were enrolled from an occupational cohort in Guinea-Bissau.

**Methods:**

CD4^+^ T-cell differentiation, activation, exhaustion, senescence, and transcription factors were assessed by polychromatic flow cytometry. Multidimensional clustering bioinformatic tools were used to identify CD4^+^ T-cell subpopulations linked to infection type and disease stage.

**Results:**

HIV-2-infected individuals had early and late-differentiated CD4^+^ T-cell clusters with lower activation (CD38^+^HLA-DR^+^) and exhaustion programmed death-1 (PD-1) than HIV-1 and HIV-D-infected individuals. We also noted that aviremic HIV-2-infected individuals possessed fewer individuals. CD4^+^ T cells with pathological signs compared to other HIV-infected groups. Still, compared to HIV-seronegative individuals, aviremic HIV-2-infected individuals had T-bet^+^ CD4^+^ T cells that showed elevated immune activation/exhaustion, and particularly the frequencies of PD-1^+^ cells were associated with a suboptimal percentage of CD4^+^ T cells.

**Conclusion:**

Increased frequencies of CD4^+^ T cells with an activated/exhausted phenotype correlate with exacerbated immunodeficiency in aviremic HIV-2-infected individuals. Thus, these findings encourage studies on the introduction of antiretroviral therapy also to individuals with aviremic HIV-2 infection.

## Introduction

Untreated HIV type 1 (HIV-1) infection is characterized by progressive decline of CD4^+^ T cells, resulting in the development of AIDS. Infection with HIV type 2 (HIV-2) may also progress to AIDS, but the likelihood is reduced (reviewed in [1]). The reason for this difference is not fully elucidated, but it is clear that the plasma viral load set-point in HIV-2-infected individuals is at least one log lower than in HIV-1-infected individuals [2,3]. Even though HIV-2 plasma viremia may emerge, and is predictive of progressive HIV-2 disease [4,5], a large proportion of HIV-2-infected individuals maintain undetectable HIV-2 plasma levels, similar to individuals with untreated aviremic HIV-1 infection (elite controllers) [2,3]. Studies have implicated that lower HIV-2 plasma levels might partly be a consequence of an efficient T-cell response, including HIV-2-specific CD4^+^ and CD8^+^ T cells with sustained functionality and specific transcriptional profiles [6–9]. Furthermore, HIV-2 can delay subsequent HIV-1 disease progression in HIV-1/HIV-2 dually (HIV-D)-infected individuals [10,11]. Therefore, studies of aviremic HIV-2-infected individuals may provide insights to how protective immunity can be harnessed and translated for future vaccine or curing strategies against both HIV-1 and HIV-2.

Despite the fact that HIV-2 represents an attenuated form of HIV, individuals infected with HIV-2 may display patterns of immune dysregulation, for example, elevated activation and exhaustion of myeloid, natural killer (NK), invariant NKT, and T cells [12–17]. Furthermore, gut disruption and microbial translocation can also be a consequence of HIV-2 infection [18,19]. Nevertheless, many of these studies have not separated aviremic from viremic HIV-2-infected individuals, and therefore large heterogeneity can be found for immune activation and other pathological characteristics. However, it was recently indicated that aviremic HIV-2-infected individuals had CD8^+^ T cells with lower immune activation and cell cycling compared to those with viremia [20]. In another study, expression levels of the programmed death-1 (PD-1) exhaustion marker on T cells were found to be different comparing aviremic and viremic HIV-2-infected individuals [15]. However, it remains largely unexplored whether specific memory CD4^+^ T-cell compartments display pathological traits in progressive HIV-2 disease without viremia.

Several lines of evidence suggest that HIV-1 elite controllers retain increased T-cell activation compared with HIV-seronegative and long-term antiretroviral therapy (ART)-treated HIV-1-infected individuals [21,22]. Studies have also demonstrated reduced T-cell activation in HIV-1 elite controllers undergoing prospective ART [23]. Moreover, some of these individuals also progress to AIDS despite undetectable viremia, and possess higher risk to develop non-AIDS-related diseases [24]. A large proportion of individuals infected with HIV-2 remain aviremic for years, but it is not clear whether these individuals have CD4^+^ T cells with markers of elevated activation and other pathological characteristics, thereby increasing their risk of AIDS and non-AIDS-related illnesses.

Here, HIV-1, HIV-2, and HIV-D-infected individuals, and also HIV-seronegative controls, were enrolled from a cohort in Guinea-Bissau [25,26]. Our aim was to describe, with new clustering in-situ tools, which memory CD4^+^ T-cell populations that were highly activated, exhausted, and transcriptionally dysregulated in these infections. Furthermore, we set out to determine whether CD4^+^ T cells with specific pathological phenotypes were elevated and associated with immunodeficiency in aviremic HIV-2 infection.

## Methods

### Study participants

The study participants were part of an occupational cohort of police officers in Guinea-Bissau [25,26] (see Supplemental Digital Content Table S1). Blood samples were obtained from HIV-1 (*n* = 33), HIV-2 (*n* = 39, of whom 26 were aviremic), or HIV-D (*n* = 13)-infected individuals, either naive to treatment or not successfully treated, that is, with viremia above the detection level. Samples from 25 HIV-seronegative individuals within the same cohort were included as controls. Informed consent was obtained from the participants and the local science coordination, the ethical committee in Guinea-Bissau, and the ethical committee at Lund University approved the study.

### Sample collection and CD4^+^ T-cell level determination

Blood samples were collected using EDTA vacutainer tubes (BD Biosciences, San Jose, California, USA, Omaha, Nebraska, USA) and Cyto-Chex BCT tubes (Streck). As previously described [27], HIV status and percentage of CD4^+^ T cells (%CD4) were determined using serology and flow cytometry, respectively. The rational for the use of %CD4 as a marker of immunodeficiency came from findings that %CD4 is a more stable disease marker than absolute CD4^+^ T-cell (CD4) count in settings with elevated pathogenic burden and comorbidities [28], and on the prior use of this marker in the studied cohort [29]. CD4 count was determined on Cyto-chex-stabilized whole blood at the Laboratory of Clinical Immunology and Transfusion Medicine, Skåne University Hospital, Lund, Sweden.

### Plasma HIV-1 and HIV-2 viral load

With minor modifications, HIV-1 and HIV-2 plasma viral loads were determined by in-house quantitiative PCR (qPCR) protocols as described [30]. Briefly, viral RNA was extracted using miRNeasy micro Kit (Qiagen, Hilden, Germany), and TaqMan qRT-PCR was performed using the Superscript III Platinum One Step qRT-PCR kit (Life Technologies, Carlsbad, California, USA). The detection limit for the viral loads was 75 RNA copies/ml plasma for HIV-1 or HIV-2 singly-infected, and 135 RNA copies/ml plasma for HIV-D-infected.

### Flow cytometry staining and analysis

**Fig. 1:**
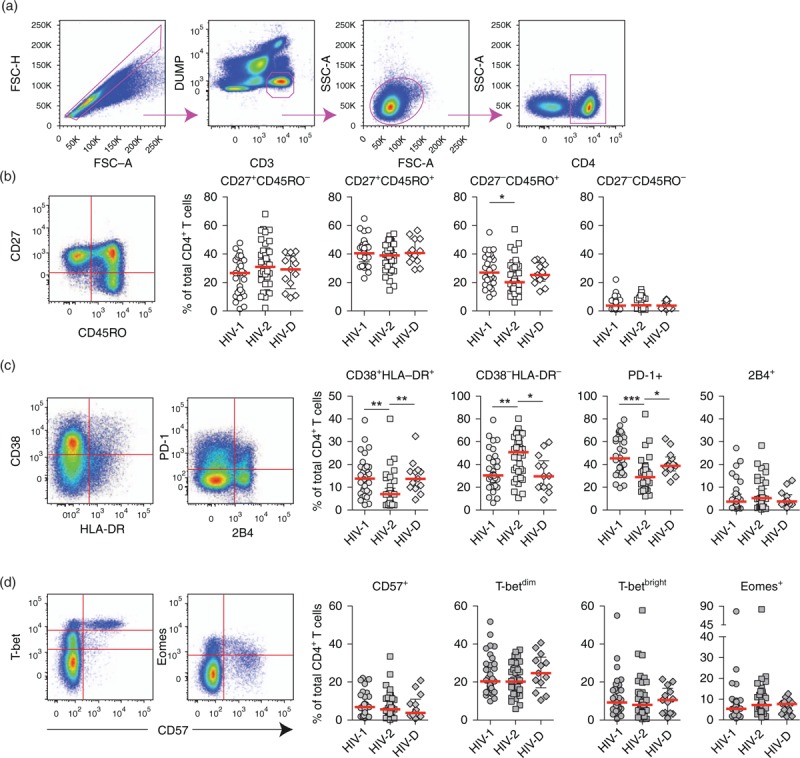
CD4^+^ T-cell populations identified by single/double expression patterns of assessed markers in HIV-1, HIV-2, and HIV-D-infected individuals.

Flow cytometry staining of markers on CD4^+^ T cells was performed on Cyto-Chex-stabilized whole blood [31]. Preanalysis was conducted to confirm that Cyto-Chex-stabilized blood generated data of all tested markers equivalent to that obtained with fresh blood samples (data not shown). Stainings of blood were adopted from protocols used for peripheral blood mononuclear cells [32]. Red blood cells were lyzed and remaining cells were stained with fluorochrome-conjugated antibodies (Supplemental Digital Content Table S2). Cells were permeabilized and fixed with the FOXP3 staining kit (eBioscience, San Diego, California, USA). Within 6 h, cells were run on a LSR Fortessa (BD Biosciences), where minimally 600 000 events were collected per run. Antibody capture beads (BD Biosciences) were used for compensation and FlowJo 8.8.7 (Tree Star, Ashland, Oregon, USA) for analyses. All manual gatings were based on fluorescence-minus-one gating strategies as described [33,34]. A typical gating strategy to distinguish CD4^+^ T cells is visualized in Fig. 1a.

(a) Gating scheme to differentiate CD4^+^ T cells. Flow and scatter plots illustrating the level of (b) memory differentiation, (c) expression of CD38, HLA-DR, PD-1, 2B4, and (d) CD57, T-bet, Eomes expression, in all HIV-infected groups. Nonparametric Kruskal–Wallis test with Dunn's multiple comparison test was conducted to compare groups; ^∗^*P* < 0.05, ^∗∗^*P* < 0.01, and ^∗∗∗^*P* < 0.001. Medians and IQR are depicted in plots. HIV-D, HIV-1/2 dually infected; IQR, interquartile range.

### Multidimensional clustering FLOw Clustering without K analysis

The FLOw Clustering without K (FLOCK) analyses were in large performed as described [35]. Thirty thousand events from every HIV-infected individual were randomly subsampled from all CD4^+^-gated population to create centroids. If a study participant had fewer than 30 000 events, all events were included. The centroids were applied to the cohorts, in which, for each individual, their events were binned into their respective closest cluster. This translates the events for each individual into frequencies of FLOCK population usage, yielding a FLOCK frequency matrix describing these frequencies for all study participants. Single-channel normalization – GaussNorm [36] – was used to correct for minor fluorescence shifts of CD45RO and CD27 expression between individuals.

### Statistical analysis

All statistical tests are described in corresponding figure legends. Statistical comparisons between two or more groups and correlations were performed using Graphpad Prism 5.0 (Graphpad Software, La Jolla, California, USA). Pie charts and permutations tests were analyzed using SPICE [37]. Clustering analysis, heat maps, and principal component analysis (PCA) were performed in R environment [38].

## Results

### CD4^+^ T-cell activation and exhaustion, but not memory differentiation, distinguish HIV-2 from HIV-1 and HIV-D infections

Previous studies have demonstrated elevated levels of T-cell pathology, such as immune activation and exhaustion, in HIV-1-infected individuals compared to HIV-2-infected and HIV-seronegative individuals [12–15,20]. However, no study has assessed these cellular characteristics comparing individuals who are singly versus dually infected with HIV-1 and HIV-2. We here conducted in-depth investigations of CD4^+^ T cells (Fig. 1a) and their simultaneous expression of markers reflecting maturation (CD45RO, CD27), activation (CD38, HLA-DR), exhaustion (PD-1, 2B4), senescence (CD57), and also transcription factors of memory differentiation and cytolytic potential (T-bet, Eomes) [39] in HIV-1, HIV-2, and HIV-D-infected individuals. We first investigated individual and dual combinations of commonly used markers distinguishing CD4^+^ T-cell subsets. Surprisingly, the frequency of CD4^+^ T-cell memory phenotypes was largely similar between the groups, in which only late-differentiated (CD45RO^+^CD27^−^) cells were elevated in HIV-1-infected compared to HIV-2-infected study participants (*P* < 0.05; Fig. 1b). The frequency of activated (CD38^+^HLA-DR^+^) cells was lower, whereas the frequency of resting (CD38^−^HLA-DR^−^) cells was higher, in HIV-2 compared to both HIV-1 (*P* < 0.001) and HIV-D (*P* < 0.05)-infected individuals. The level of PD-1-expressing cells followed similar trends and was reduced on CD4^+^ T cells in HIV-2 compared to both HIV-1 (*P* < 0.001) and HIV-D (*P* < 0.05) infections (Fig. 1c). Level of 2B4 expressing cells, and also CD57, T-bet, and Eomes, was similar between all the groups (*P* > 0.05), implicating that markers of late/terminal differentiation and senescent CD4^+^ T cells are not hallmarks that distinguish HIV-1 from HIV-2 infection (Fig. 1d).

### Clustering-based analysis define four separate memory CD4^+^ T-cell populations that are elevated in HIV-1-infected individuals

**Fig. 2:**
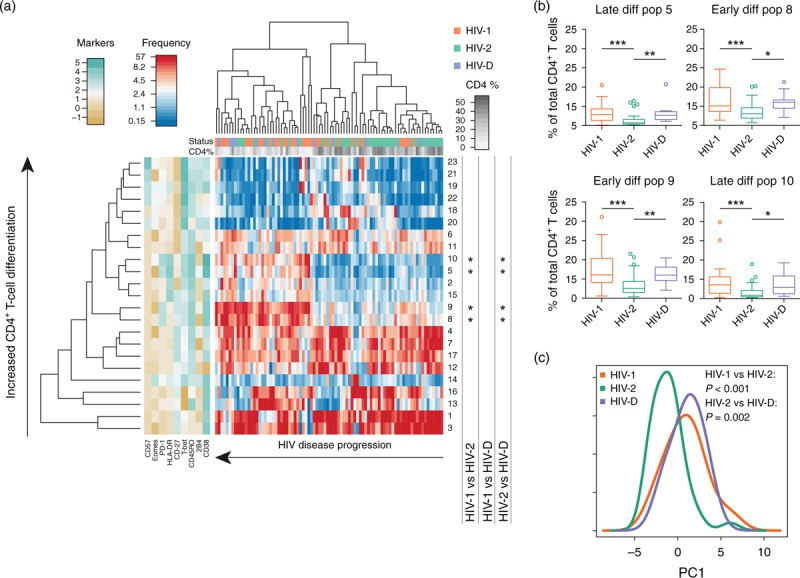
CD4^+^ T-cell populations identified by expression patterns of all assessed markers in HIV-1, HIV-2, and HIV-D-infected individuals evaluated by FLOCK analysis.

In order to combine the measurement of all assessed markers, we used a multidimensional clustering method, FLOCK [35,40], to delineate whether specific CD4^+^ T-cell clusters were differently expressed between the three HIV-infected groups. Using this approach, we identified 23 unique CD4^+^ T-cell populations after including CD4^+^ T cells from all HIV-infected study participants. By employing unsupervised hierarchical clustering analysis on the 23 unique populations, we identified clustering that was particularly dependent on the CD4^+^ level and HIV status (horizontal dendrograms of the individuals) and memory differentiation (vertical dendrograms of the FLOCK populations) (Fig. 2a). Furthermore, two memory clusters were primarily found to be elevated within the HIV-1 and HIV-D-infected individuals compared to the HIV-2-infected individuals: one early-differentiated (CD27^+^CD45RO^+^) cluster with increased CD38 and PD-1, but low HLA-DR levels and with variable expression levels of 2B4 and Eomes (populations 8 and 9), and a late-differentiated (CD27^−^CD45RO^+^) cluster with elevated levels of CD38, HLA-DR, and PD-1, and also variable expression of 2B4 and Eomes (populations 5 and 10) (Fig. 2b; Supplemental Digital Content Fig. S1). These two populations also demonstrated intermediate expression levels of T-bet. Finally, a PCA on all 23 FLOCK population frequencies were performed. PCA is an unsupervised statistical method for reducing data dimensionality while retaining the vital variation in fewer informative variables. Specifically one component (PC1) of the PCA was significantly lower for individuals infected with HIV-2 compared to HIV-1 (*P* < 0.001) and HIV-D (*P* < 0.01) (Fig. 2c).

(a) The FLOCK frequency matrix is visualized (center red/blue) using a heat map representation. The rows denote the FLOCK populations and the columns represent HIV-infected individuals. The color of each tile denotes the population frequency for the specific FLOCK population in the specific patient. Unsupervised hierarchical clustering was performed on both the rows and columns of the FLOCK frequency matrix. The heat map on the (green/brown) left-hand side shows the average mean fluorescent intensities (MFIs) for each FLOCK population. The top two bars show the HIV status of each individual and their corresponding %CD4. The populations found to be significantly different between HIV-1, HIV-2, and HIV-D are denoted by the asterisk on the right-hand side of the main heat map. A bar plot representation of the frequencies of these four FLOCK populations, late differentiated (Diff) population (Pop) 5 and 10 (CD38^++^HLA-DR^+^PD-1^+^T-bet^+^) and early diff Pop 8 and 9 (CD38^++^HLA-DR^−^PD-1^+^T-bet^+^) can be seen in (b). A PCA was performed on all FLOCK populations where the first principle component (PC1) is used to illustrate the population variation of each HIV-infected group (HLA-DR^−^) through a histogram in (c). Nonparametric Kruskal–Wallis test with Nemenyi's multiple comparison test were conducted to compare group differences; ^∗^*P* < 0.05, ^∗∗^*P* < 0.01, and ^∗∗∗^*P* < 0.001. Whiskers in the box plots are based on Tukey distribution. Kolmogorov–Smirnov tests were used in the PCA histogram to test whether two HIV-infected groups were drawn from different distributions. FLOCK, FLOw Clustering without K; HIV-D, HIV-1/2 dually infected; PCA, principal component analysis.

### Viremic HIV-2-infected individuals possess higher CD4^+^ T-cell activation and exhaustion than aviremic individuals

**Fig. 3:**
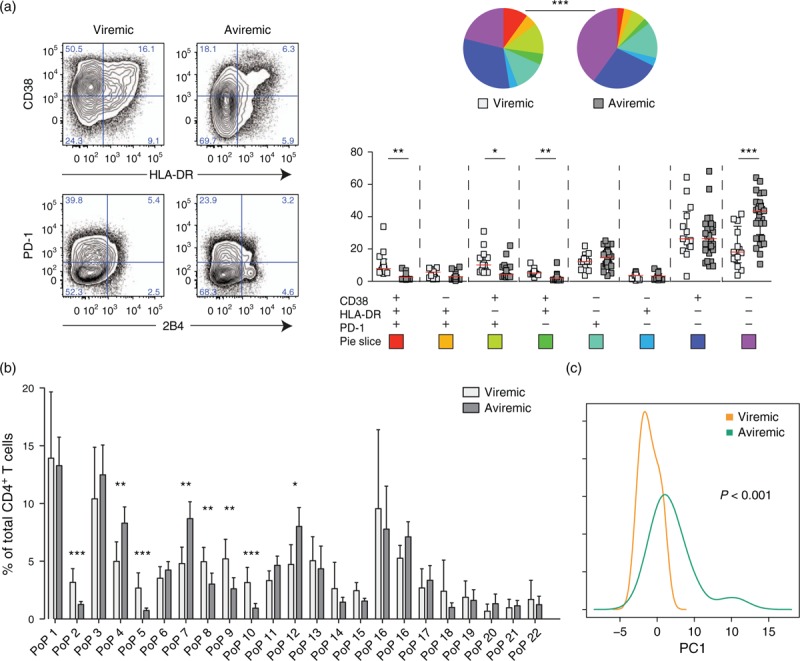
Frequencies of CD4^+^ T-cell subpopulations comparing viremic and aviremic HIV-2-infected individuals.

Despite that HIV-2-infected study participants demonstrated lower levels of CD4^+^ T-cell activation and exhaustion, we found a large heterogeneity in this group where some individuals clustered together with the HIV-1-infected group (Fig. 2). Therefore, we next analyzed whether individuals with detectable (viremic, *n* = 13) versus undetectable (aviremic, *n* = 26) HIV-2 RNA plasma levels showed differences between the measured CD4^+^ T-cell parameters. We first assessed single and dual-marker combinations of all measured parameters. In line with our previous observations, no major differences were observed in regard to naive/memory phenotype distributions, T-box transcription factors, CD57, and 2B4 expression (data not shown). However, the co-expression pattern of CD38, HLA-DR, and PD-1 were statistically different between the viremic versus aviremic HIV-2-infected groups (permutation test; *P* < 0.001) (Fig. 3a). Using Boolean gating principles, we found that the frequency of CD38^+^HLA-DR^+^PD-1^+^ (*P* < 0.01), CD38^+^HLA-DR^−^PD-1^+^ (*P* < 0.05), and CD38^+^HLA-DR^+^PD-1^−^ (*P* < 0.01) CD4^+^ T cells were elevated within viremic individuals, whereas the nonactivated/nonexhausted state (CD38^−^HLA-DR^−^PD-1^−^) was higher within aviremic study participants (*P* < 0.001; Fig. 3a). Strikingly, HIV-2 viremic individuals possessed similar CD4^+^ T-cell activation and exhaustion as the HIV-1 and HIV-D-infected groups (Fig. 2) despite a 1.3 log lower plasma viral load than the HIV-1-infected individuals (see Supplemental Digital Content Table S1).

Distribution of populations defined by (a) expression of activation/exhaustion markers (CD38, HLA-DR, and PD-1), (b) FLOCK analysis, and (c) PCA histogram comparing viremic and aviremic HIV-2-infected groups. SPICE analysis: median and IQR are shown for all scatter plots/columns and Mann–Whitney tests were performed to compare outcomes between groups; ^∗^*P* < 0.05, ^∗∗^*P* < 0.01, and ^∗∗∗^*P* < 0.001. Significant differences were identified for FLOCK population (Pop) 2 (late differentiated CD38^+^HLA-DR^+^PD-1^−^T-bet^+^); pop 4/7 (early differentiated CD38^+^HLA-DR^−^PD-1^−^T-bet^-/+^); pop 5/10 (late differentiated CD38^++^ HLA-DR^+^PD-1^+^T-bet^+^); pop 8/9 (early differentiated CD38^++^HLA-DR^−^PD-1^+^T-bet^+^), and pop 12 (intermediate differentiated CD38^−^HLA-DR^−^PD-1^−^T-bet^+^). Permutation test was performed between the pie charts of CD38, HLA-DR, and PD-1 combinations. Kolmogorov–Smirnov tests were used in the PCA histogram to test whether two HIV-2-infected groups were drawn from different distributions. FLOCK, FLOw Clustering without K; HIV-D, HIV-1/2 dually infected; PCA, principal component analysis.

Next, FLOCK was adapted to identify specific CD4^+^ T-cell clusters that differed between the viremic versus aviremic HIV-2-infected patients. We found that only early or late (CD45RO^+^) differentiated CD4^+^ T-cell individuals. populations with increased CD38 expression, and variable levels of HLA-DR, PD-1, 2B4, CD27, Eomes, and T-bet, were elevated within viremic individuals (populations 2, 5, 8, 9, and 10) (Fig. 3b). Similarly, only memory (CD45RO^+^) CD4^+^ T-cell populations, but with lower CD38, HLA-DR, and PD-1 levels, were increased in aviremic individuals (populations 4, 7, and 12) (Fig. 3b). Also, here the PCA confirmed that the overall combination of all FLOCK populations were significantly different, based on PC1 score (*P* < 0.001), between the viremic and aviremic HIV-2-infected individuals (Fig. 3c).

### Elevation of CD4^+^ T-cell activation/exhaustion in aviremic HIV-2 infection

**Fig. 4:**
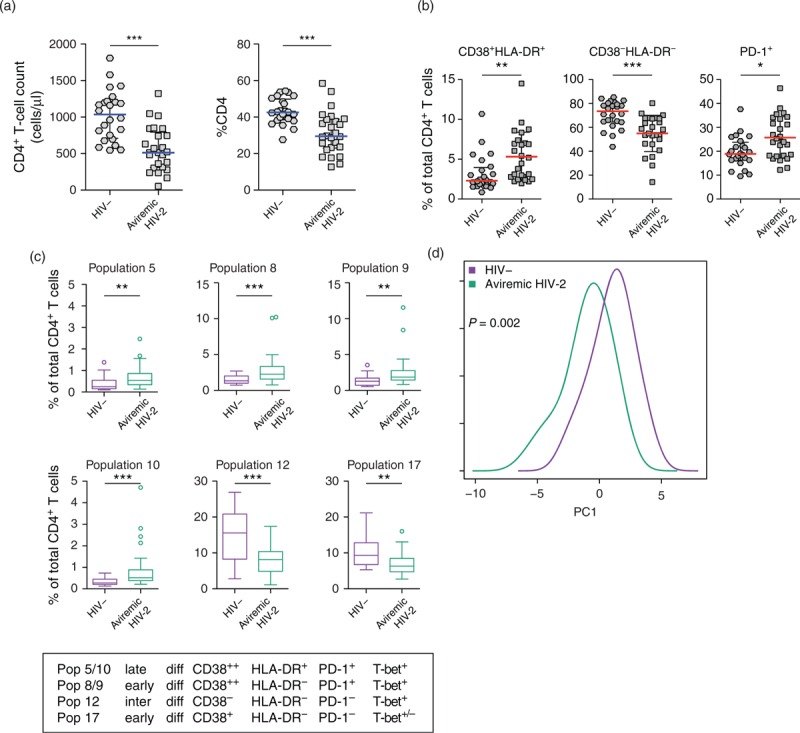
Frequencies of CD4^+^ T-cell subpopulations comparing aviremic HIV-2-infected and HIV-seronegative individuals.

Although the aviremic HIV-2-infected study participants possessed lower combined and single measurements of CD4^+^ T-cell pathology than those with viremic HIV-2 infection, we found that these individuals had 48% fewer absolute count CD4^+^ T cells (median 513 versus 1037 cells/μL; *P* < 0.001) and %CD4 was reduced with 30% (mean 29.5 versus 42.6%; *P* < 0.001) compared with HIV-seronegatives from the same cohort (Fig. 4a). We therefore hypothesized that reduced CD4^+^ T-cell levels were associated with altered expression of markers indicating CD4^+^ T-cell pathology. No T-box transcriptional or memory differentiation differences were observed (data not shown), but again both CD38^+^HLA-DR^+^ (*P* < 0.01) and PD-1^+^ (*P* < 0.05) frequencies were significantly elevated among CD4^+^ T cells in aviremic HIV-2-infected individuals (Fig. 4b). Interestingly, the reduction of resting CD4^+^ T cells in the aviremic individuals was particularly strong (*P* < 0.001) (Fig. 4b). Similarly, FLOCK analysis identified that early and late-differentiated CD4^+^ T-cell clusters (populations 5, 8, 9, and 10) commonly displaying increased CD38 and PD-1 levels were elevated in the aviremic HIV-2-infected individuals (Fig. 4c). In contrast, memory CD4^+^ T cells with no expression of HLA-DR and PD-1, and variable levels of CD38, 2B4, and T-bet (populations 12 and 17) were higher within HIV-seronegative study participants (Fig. 4c), demonstrating an imbalance of resting memory CD4^+^ T cells in aviremic individuals with HIV-2 infection. The PC1 score on all FLOCK population frequencies demonstrated significant differences between HIV-seronegative and HIV-2 aviremic individuals (*P* < 0.01; Fig. 4d).

(a) CD4^+^ T-cell levels (absolute CD4 count and %CD4), (b) distribution of populations defined by expression of activation/exhaustion markers (CD38, HLA-DR, and PD-1), or (c) FLOCK analysis, and (d) PCA histogram, comparing aviremic HIV-2-infected and HIV-seronegative groups. Mann–Whitney tests were performed to determine significant differences between the populations; ^∗^*P* < 0.05, ^∗∗^*P* < 0.01, and ^∗∗∗^*P* < 0.001 (median and IQR). Significant differences were identified for FLOCK population (Pop) 5/10 (late differentiated CD38^++^HLA-DR^+^PD-1^+^T-bet^+^); Pop 8/9 (early differentiated CD38^++^ HLA-DR^−^PD-1^+^T-bet^+^); Pop 12 (intermediate differentiated CD38^-^ HLA-DR^−^PD-1^−^T-bet^+^), and Pop 17 (early differentiated CD38^+^HLA-DR^−^PD-1^−^T-bet^−/+^). Kolmogorov–Smirnov tests were used in the PCA histogram to test whether two HIV-infected groups were drawn from different distributions. FLOCK, FLOw Clustering without K.

### Combined CD38, HLA-DR, and PD-1 expression is primarily associated with lower %CD4 and CD4 count in aviremic HIV-2 infection

**Fig. 5:**
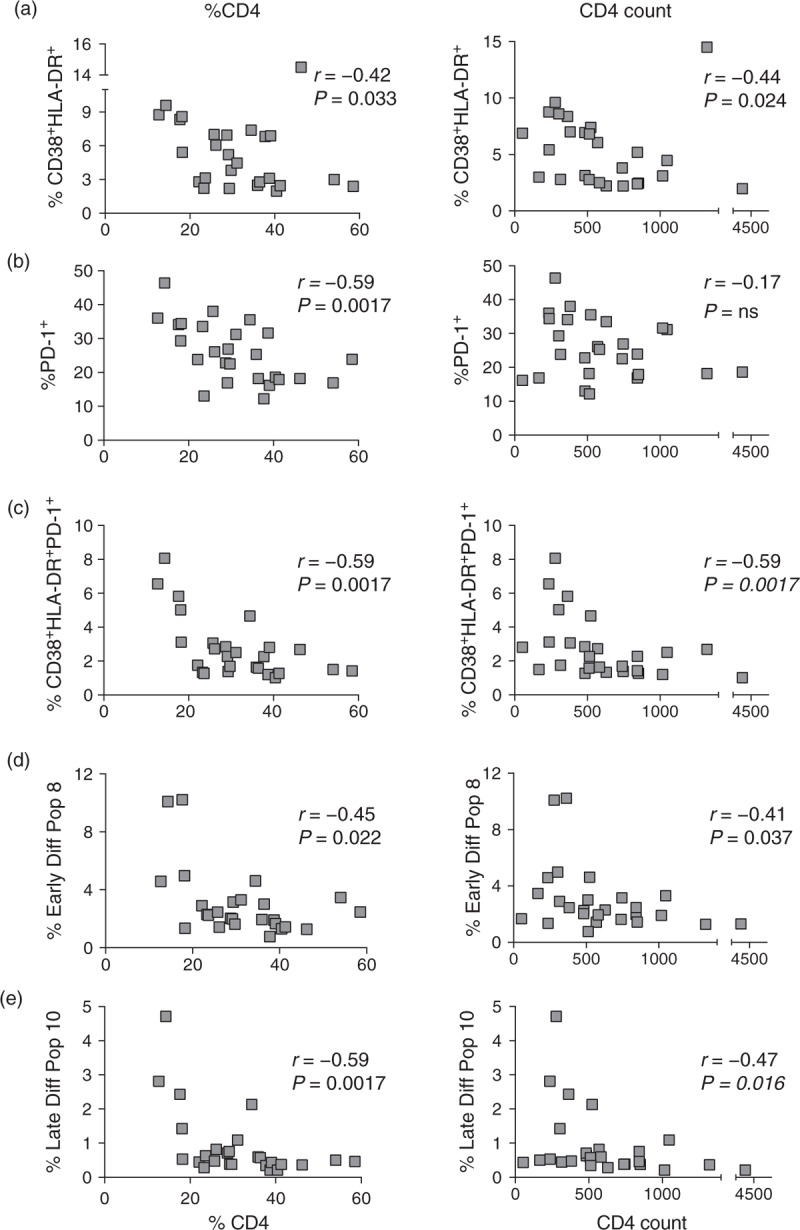
Frequencies of CD4^+^ T-cell subpopulation in correlation to levels of CD4^+^ T cells among aviremic HIV-2-infected individuals.

We finally evaluated whether the suboptimal CD4^+^ levels in the aviremic HIV-2-infected individuals were associated with heightened CD4^+^ T-cell activation/exhaustion or lower levels of resting CD38^−^HLA-DR^−^ cells. Despite the fact that aviremic HIV-2-infected individuals demonstrated lower frequencies of CD38^−^HLA-DR^−^ cells than HIV-seronegative controls, no significant correlation was observed between the frequency of resting CD4^+^ T cells and %CD4 or CD4 count (data not shown). Percentage of CD38^+^HLA-DR^+^ CD4^+^ T cells was instead inversely associated with both %CD4^+^ (*r* = -0.42, *P* < 0.05) and CD4 count (*r* = −0.44, *P* < 0.05) (Fig. 5a). In contrast, the frequency of PD-1^+^ CD4^+^ T cells correlated inversely only with %CD4 (*r* = −0.59, *P* < 0.01), and not CD4 count (Fig. 5b). In line with the Boolean gating analysis (Fig. 3a) was the triple combination of CD38, HLA-DR, and PD-1 indicative of disease severity, both assessed as %CD4 and CD4 count, in aviremic HIV-2 infection (*r* = −0.59, *P* < 0.01; Fig. 5c). After plotting the frequencies of FLOCK-mediated CD4^+^ T-cell clusters, we found that also the early differentiated CD38^++^HLA-DR^−^PD-1^+^T-bet^+^ population 8 correlated inversely with both %CD4 and CD4 count (*r* = −0.45, *P* < 0.05; and *r* = 0.41, *P* < 0.05, respectively; Fig. 5d). Still, it was particularly the late differentiated CD38^++^HLA-DR^+^PD-1^+^T-bet^+^ population 10, with the highest PD-1, CD38, and HLA-DR fluorescent intensity levels, that correlated with low %CD4 and CD4 counts in aviremic HIV-2 infection. Collectively, our results show that T-bet^+^ memory CD4^+^ T cells with specific pathological phenotypes, including a combination of elevated CD38, HLA-DR, and PD-1, are associated with reduced CD4^+^ T-cell levels in aviremic HIV-2 infection.

Correlations between percentage CD4^+^ T cells or absolute CD4^+^ T-cell count and (a) CD38^+^HLA-DR^+^, (b) PD-1^+^, (c) CD38^+^HLA-DR^+^PD-1^+^, (d) FLOCK population (Pop) 8 (early differentiated CD38^++^HLA-DR^−^PD-1^+^T-bet^+^), and (e) Pop 10 (late differentiated CD38^++^HLA-DR^+^PD-1^+^T-bet^+^) CD4^+^ T cells among HIV-2 aviremic individuals. The Spearman rank nonparametric test was used for correlations analysis. FLOCK, FLOw Clustering without K.

## Discussion

Deeper knowledge of HIV-infected individuals controlling their virus might potentially lead to novel insights into how protective immunity could be translated in future therapeutic cure or vaccine approaches. Studies investigating whether aviremic HIV-2 infection is associated with increased CD4^+^ T-cell pathology remain, however, limited [12,15,20], and no study, to our knowledge, has shown whether immunodeficiency in aviremic HIV-2-infection is linked to altered memory CD4^+^ T-cell pathology. By the support of an interdisciplinary approach, we here, in side-by-side analysis of blood samples from HIV-1, HIV-2, or HIV-D-infected individuals, identified that HIV-2-infected aviremic individuals demonstrate the lowest frequencies of CD4^+^ T cells with an activated/exhausted phenotype. Still, memory CD4^+^ T cells expressing these markers were significantly elevated in aviremic HIV-2-infected individuals, and more importantly, were inversely correlated with lower CD4^+^ T-cell levels, compared to HIV-seronegative individuals from the same cohort.

Intriguingly, we noted that the strongest correlation to reduced %CD4^+^ T cells in HIV-2 aviremic infection was linked more to accumulation of PD-1^+^ and less to CD38^+^HLA-DR^+^ cells. Of note, HIV-1-infected individuals demonstrated a different relationship, in which PD-1^+^ (*r* = −0.74, *P* < 0.001) and CD38^+^HLA-DR^+^ (*r* = −0.71, *P* < 0.001) cells demonstrated similar correlation coefficients to reduced %CD4 (data not shown). These data suggest that PD-1, partly independently of the activation status, hinders normal immune homeostasis of memory CD4^+^ T cells in aviremic HIV-2 infection, an assumption also supported by the difference in %PD-1^+^ cells observed between our controls and aviremic HIV-2-infected individuals. Tendeiro *et al.*[15] did not find any difference in PD-1 expression between aviremic individuals and controls. The dissimilar result might be due to the usage of different anti-PD-1 antibody clones and the interpretation of the flow data, based on mean fluorescent intensity (MFI) [15] or cell frequencies. In addition, a significant proportion (77%) of the aviremic HIV-2-infected individuals in our study had suboptimal CD4^+^ T cell levels, below the interquartile range of the HIV-seronegative individuals. Interestingly, strong links between elevated frequency of cells expressing PD-1, in combination with CD38 and HLA-DR, was noted with reduced levels of both %CD4 and CD4 count. However, when analyzing %PD-1^+^ cells separately, there was only an association with %CD4, in line with our earlier observations that CD4/CD8 ratio and %CD4 correlate better with pathological T-cell phenotypes than CD4 count [33]. The simultaneous assessment of all markers and specific CD4^+^ T-cell populations using FLOCK analysis also revealed that PD-1-expressing populations, primarily the activated late-differentiated, were associated with low CD4^+^ T-cell levels in aviremic HIV-2 infection. The intermediate to high T-bet levels furthermore support that increased exhaustion, and partly activation, is particularly associated with immunodeficiency during aviremic HIV-2 infection.

Similarly to our analysis of aviremic HIV-2-infected individuals, studies have demonstrated that HIV-1 elite controllers demonstrate elevated T-cell activation that is associated with progressive disease [21,22]. A recent study reported, however, that PD-1 MFI levels on CD4^+^ T cells were not different between HIV-1 elite controllers and HIV-seronegative individuals, although PD-1 was highly predictive of failing CD4^+^ T-cell recovery after ART initiation [41]. The CD4+ T-cell levels of the HIV-2 aviremic individuals participating in the current study varied widely and were significantly lower than in the HIV-seronegative individuals. Thus, it seems likely that the aviremic HIV-2-infected individuals in our study partly differ from American/European HIV-1 elite controllers, in whom CD4^+^ T cell levels are relatively preserved [21,22,41]. The reason for these differences may be multifactorial, including type of HIV infection (HIV-1 versus HIV-2) and geographical location. Another difference could be duration of HIV infection. Because the majority of the HIV-2 aviremic individuals in our study had been diagnosed more than 15 years before the sampling (data not shown), one can assume that they had been infected for decades. Progressively increased expression of PD-1 on both CD4^+^ and CD8^+^ T-cell subsets have been shown by longitudinal studies of untreated HIV-1 infection [41], and could likely be a component of failed CD4^+^ T-cell regeneration. Similarly to HIV-1 elite controllers, HIV-2 aviremic individuals could also have ongoing virus replication. Ultrasensitive PCR analyses and virus phylogenetic studies support ongoing low-grade virus replication in HIV-1 elite controllers [42,43]. Such studies have not yet been performed in HIV-2 aviremic infections, but it can be anticipated that ultrasensitive assays will detect low-grade viremia in a substantial proportion of HIV-2-infected individuals now classified as aviremic, as indicated by a digital droplet PCR protocol [44]. It is, however, intriguing that proviral loads in HIV-2-infected individuals have been reported to be similar to that of HIV-1-infected individuals, despite the large difference in plasma viral load [45]. It has also been shown by Soares *et al.*[46] that HIV-1 and HIV-2-infected individuals matched for CD4^+^ exhibit similar gag mRNA levels, suggesting that viral transcription occurs, despite low plasma viral load in HIV-2 infection, which might fuel the disease progression in aviremic HIV-2 infection.

In line with other studies, we also found that CD4^+^ T-cell activation [12,20] and PD-1 expression [15] were elevated in viremic compared to aviremic HIV-2 infection. Here, clustering analysis revealed that it was CD45RO^+^ (memory) CD4^+^ T-cell populations with elevated CD38 expression that accumulated in the viremic HIV-2-infected individuals. Several of these highly activated populations also expressed intermediate to high levels of T-bet, which implicate that many of the changes could potentially occur in T-helper 1 polarized cells (reviewed in [47]). Furthermore, the elevated CD38 levels in HIV-2 viremic individuals stands in contrast to the dominance of PD-1 expression during immunodeficiency in aviremic HIV-2 infection, and may suggest that markers of activation and exhaustion appear with different kinetics during the HIV-2 disease course, and that T-cell activation is more closely related to the extent of viremia.

In summary, we here describe that aviremic HIV-2-infected individuals in general demonstrate lower expression levels of pathological CD4^+^ T-cells markers than viremic HIV-1 and HIV-2-infected individuals. However, aviremic HIV-2-infected individuals still display specific memory CD4^+^ T-cell compartments with elevated activation and exhaustion compared with HIV-seronegative individuals. Taken together, we believe these findings should be the incentive for future studies/trials examining whether CD4^+^ T-cell decline and immune activation/exhaustion could be reversed by administration of ART also to HIV-2 aviremic individuals.

## Acknowledgements

The listed authors and the members of the Sweden Guinea-Bissau Cohort Research (SWEGUB CORE) group, including Babetida N’Buna, Antonio Biague, Ansu Biai, Cidia Camara, Joakim Esbjörnsson, Marianne Jansson, Sara Karlson, Jacob Lopatko Lindman, Patrik Medstrand, Fredrik Månsson, Hans Norrgren, Angelica A. Palm, Gülsen Özkaya Sahin, and Zacarias Jose da Silva, are indebted to the staff of the Police Clinics and the National Public Health Laboratory (LNSP) in Bissau, Guinea-Bissau.

Author contributions: M.B. designed and conducted the flow cytometric analyses, interpreted the data, and drafted the manuscript; J.F. designed, conducted, and summarized the bioinformatics analyses; O.L., M.R.B., M.N., J.T., H.N., P.M., and A.C.K. contributed substantially to the study conception and design, results interpretation, and critically reviewed the manuscript; A.J.B. was medically responsible for the study participants; M.J. was the principal investigator of the study.

Funding: This study was supported by grants from The Swedish Research Council (M.B., A.C.K., H.N., P.M., and M.J.); the Crafoord Foundation (M.J.); Physicians Against AIDS Research Foundation (M.B. and A.C.K.); Karolinska Institutet (A.C.K.) Magnus Bergvalls foundation (A.C.K.); the Argentinean national research council (CONICET) (M.N.).

### Conflicts of interest

The authors declare no conflicts of interests

## Supplementary Material

Supplemental Digital Content
