# Supplementary material for: CD4+ T cells with an activated and exhausted phenotype distinguish immunodeficiency during aviremic HIV-2 infection
Source: AIDS. 2016 Sep 28;30(16):2415–26. doi: 10.1097/QAD.0000000000001223 (PMC5051526; doi:10.1097/QAD.0000000000001223)
Supplement: Supplemental Digital Content [file aids-30-2415-s001.pdf]

Table S1) Characteristics of study participants

	HIV-1	HIV-2	*HIV-2 viremic*	*HIV-2 aviremic*	HIV-D	HIV seronegative
Numbers (females/males)	33 (14/19)	39 (11/28)	*13 (2/11)*	*26 (9/17)*	13 (3/10)	25 (10/15)
Age in years *	46 (39-52)	53 (47-58)	*53 (46-57)*	*54 (48-58)*	46 (43-52)	48 (40-55)
% CD4+ T cells*	10.3 (6.4-19.9)	25.0 (15.2-35.9)	*14.9 (12.2-21.1)*	*29.5 (23.0-38.8)*	12.4 (11.0-20.9)	42.3 (38.0-49.4)
CD4+ T cell Count, cells/µl*	193 (101-405)	482 (236-746)	236 (194-495)	518 (312-843)	270 (151-350)	1087 (724-1211)
Viral load, RNA copies/ml*	12009 (2535-25938)	<75 (<75-270)	*891 (232-4405)*	*<75*	2530 (900-14620)	NA

Table S2: FACS antibody staining panel

Antibody	Conjugate	Clone	Company
CD3	APC H7	SK7	BD Biosciences
CD14	V500	M5E2	BD Biosciences
HLA-DR	BV605	G46	BD Biosciences
CD19	BV711	SJ25C1	BioLegend
CD27	BV785	O323	BioLegend
CD38	PE-Cy7	HIT2	BioLegend
PD-1	BV421	EH12.2H7	BioLegend
CD45RO	ECD	UCHL1	Beckman Coulter
CD57	FITC	NC1	Beckman Coulter
2B4	PE-Cy5	C1.7	Beckman Coulter
Eomes	EF660	WD1928	eBioSciences
T-bet	PE	4B10	eBioSciences
CD4	PE-Cy5.5	S3.5	Life Technologies
Live/Dead	Aqua Blue		Life Technologies

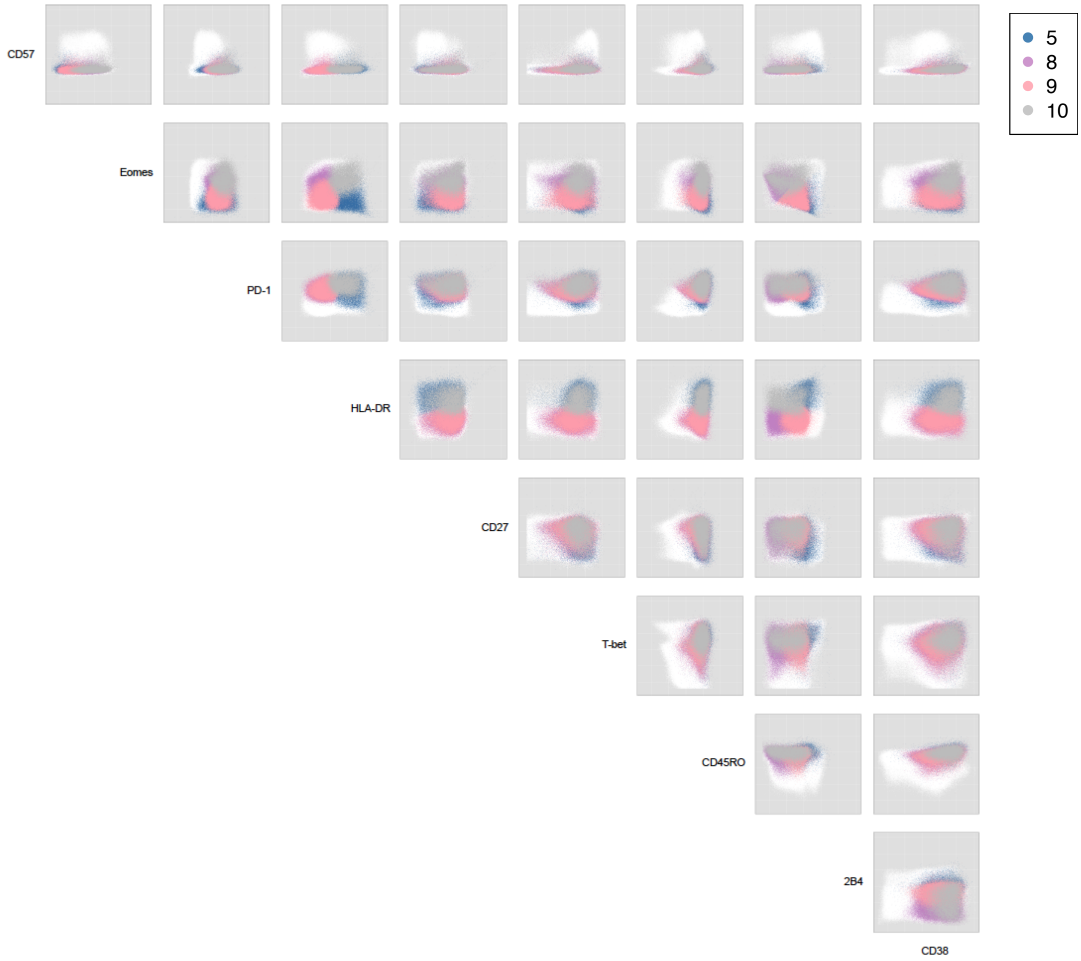

Supplementary Figure S1: Significant elevated FLOCK populations in HIV-infected subjects. CD4+ T cell expression pattern of all four "pathological" FLOCK populations that significantly differed between the HIV-1 and HIV-2-infected subjects. The FLOCK population numbers are listed in the top right corner.
